# Animal rabies prevention and control in Shanghai, China: policies and practices, 2011–2025

**DOI:** 10.3389/fpubh.2025.1729143

**Published:** 2026-01-05

**Authors:** Tingyi Zhu, Xiaojing Chang, Xiujuan Wu, Luming Xia, Xiaoying Zhu, Jiuchao Zhu, Zengqiang Li, Weifeng Chen, Yufeng Fan, Yanting Tang, Jian Liu, Hongjin Zhao

**Affiliations:** Shanghai Animal Disease Prevention and Control Center, Shanghai, China

**Keywords:** compulsory vaccination, dog management, one health, postexposure prophylaxis, rabies

## Abstract

**Background:**

Rabies remains a public health issue in Shanghai, with human cases reported since 2006, necessitating sustained multi-faceted control.

**Strategies:**

Key measures include mandatory canine vaccination, increased access to vaccination services (19–431 by 2025), management of free-roaming animals, and public education campaigns.

**Challenges and perspective:**

Challenges persist—particularly the low vaccination coverage among free-roaming animals. Future priorities involve developing oral rabies vaccines for free-roaming animals, enhancing wildlife rabies surveillance, expanding insurance coverage for postexposure prophylaxis, and strengthening cross-sector collaboration through the One Health framework.

**Conclusion:**

Shanghai’s integrated strategies have reduced human rabies. Achieving the 2030 zero-death goal requires closing gaps in free-roaming animal vaccination and postexposure prophylaxis access.

## 1 Background

Rabies is a zoonotic disease caused by the rabies virus (*lyssavirus*) and can infect all kinds of mammals, including dogs, cats, bats, foxes, raccoons, and livestock (1–9). Globally, rabies is responsible for approximately 59,000 deaths each year, with the majority of cases occurring in Asia and Africa (10–13). In China, the incidence of human rabies has significantly declined in recent years (8, 10, 14–18). However, provinces such as Hunan, Henan, and Guangxi continue to report relatively high case numbers (14, 16–18). According to official data from the Chinese Center for Disease Control and Prevention and the Shanghai Municipal Health Commission, China reported a total of 487 human rabies cases in the past 3 years, with only 1 case occurring in Shanghai (19, 20). In Shanghai, human rabies infections have been reported continuously since 2006 (13, 21). Nevertheless, due to effective control measures, no cases were reported in 2015 and between 2019 and 2021 (21).

Despite its near 100% fatality rate, rabies is entirely preventable (10, 13, 22–24). The combination of mass dog vaccination and postexposure prophylaxis (PEP) has proven highly effective in reducing rabies morbidity in many countries (3, 11, 22, 25–27). Public education and strengthened surveillance systems also play critical roles (15, 22, 25). Globally, more than 99% of human rabies cases are caused by dog bites—a trend that holds true in China as well (2, 6, 28–31). As such, mass dog vaccination remains the most effective strategy for eliminating human rabies (2, 7, 23, 32).

In Shanghai, significant efforts have been made to control rabies. This report summarizes recent policies and evaluates their effectiveness to provide insights for other provinces and international communities facing high rabies incidence.

## 2 Strategies

### 2.1 Compulsory vaccination policies and canine management regulations

Shanghai has implemented various dog-control regulations. The *“Administrative measures of Shanghai Municipality on dogs”* (1985, formalized in 1993) were superseded by the *“Regulations of Shanghai Municipality on Dog Management”* in 2011 (33). Alongside the *“Shanghai Animal Epidemic Prevention Regulations*,*”* these regulations clarify the requirements for dog registration, dog owner conduct standards, compulsory rabies vaccination and dog licensing (34). Since then, the enforcement of compulsory vaccinations has progressively increased (2, 33). Since 2022, residents have been able to apply for, renew, and modify information on dog registration certificates via the *“Suishenban”* online portal, markedly improving the accessibility and efficiency of licensing procedures.

### 2.2 Animal rabies vaccination points management

**Figure 1:**
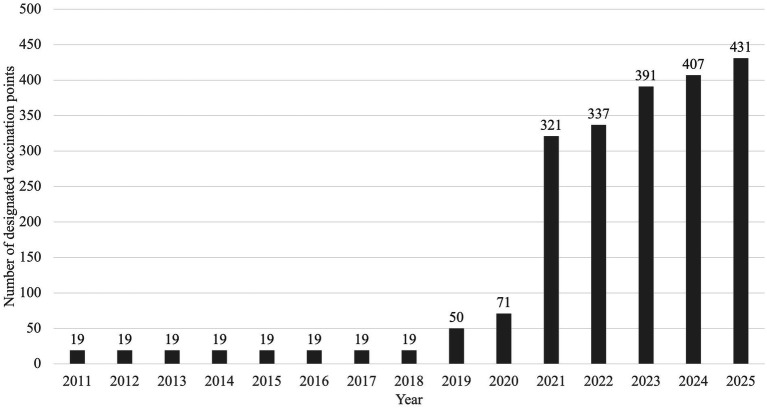
The number of designated vaccination points in Shanghai, from 2011 to 2025.

Shanghai conducts an annual designation process for animal rabies vaccination facilities. Newly applied facilities undergo rigorous vetting of qualifications, equipment, and management systems. Vaccine pricing is standardized citywide at 60 RMB per injection, and personnel at designated points receive annual training. Additionally, these points must assist the Shanghai Animal Disease Prevention and Control Center in rabies epidemiological investigations and submit monthly surveillance reports. The Municipal Agriculture Committee conducts biannual evaluations at all designated points, assessing administrative performance, antibody seropositivity rates, vaccination volumes, and surveillance cooperation. Facilities scoring below 80 are excluded (2, 33). According to data collected by our center, a notable expansion occurred in the number of designated vaccination points, which grew from 19 to 431 by August 2025 (Figure 1). Information on vaccination points is accessible via “*Suishenban*.”

To improve access in suburban areas, Shanghai conducts an annual mass vaccination campaign each spring, in addition to year-round services at designated points. These campaigns involve bringing dogs to designated locations or home visits by veterinary teams. Since July 2012, rural dog registration fees have been waived, and districts such as Minhang, Songjiang, Jinshan, and Jiading subsidize vaccination costs (34, 35). As a result, coverage and seropositivity have improved significantly, with antibody positivity exceeding 90% (2, 36).

The expansion of efforts has led to a marked increase in immunization. Our center conducts annual monitoring of the administration of rabies vaccines and the levels of rabies antibodies in canines and felines. The vaccination rate of household dogs now exceeds 85%, while seropositivity generally remains above 80% (2, 33–35). The Annual data is shown in Table 1.

**Table 1:** The rabies antibody seropositivity in household dogs in Shanghai, from 2011 to 2024.

Year	Antibody seropositivity (%)
2011	81.3
2012	82.7
2013	80.8
2014	87.4
2015	85.2
2016	80.7
2017	79.2
2018	86.9
2019	85.6
2020	88.5
2021	94.4
2022	91.0
2023	78.9
2024	84.2

### 2.3 Free-roaming animal management and control

Pet ownership is becoming increasingly popular in contemporary society. However, this trend has contributed to large free-roaming populations. Shanghai has more than 400,000 free-roaming cats (37). To address this concern, Shanghai has launched trap-neuter-return (TNR) pilot programs across various communities (38). These programs have proven effective in curbing free-roaming cat populations within a few months. Residents’ satisfaction rate with the TNR program exceeded 95% (38–40). Multiple Shanghai government departments have collaborated to provide joint guidance for TNR programs, including the Disease Control Center, Spiritual Civilization Office, Subdistrict Administrations, Agriculture Committee, and Neighborhood Committees (38).

Abandoned and lost pet dogs are the primary cause of the free-roaming dog populations in Shanghai (2). Similarly, the rise in pet dog ownership has led to a continuous increase in free-roaming dog populations. Shanghai has systematically enhanced its free-roaming dog management in recent years, with the Municipal Public Security Bureau responsible for dog shelter management (2). Dogs are available for adoption at the shelter, where euthanasia is performed on those in severely compromised health. Our center collaborates with the Public Security Bureau to conduct quarterly disease surveillance for newly sheltered dogs.

Concurrently, reports of wildlife-transmitted rabies have gradually increased in China. Contact between wild animals (such as raccoon dogs, badgers, and weasels), free-roaming dogs, and cats within communities increases the risk of rabies transmission. Shanghai has conducted surveys of raccoon dog populations and their distribution over multiple years, confirming their presence in at least 260 citywide communities (37).

### 2.4 Public education

According to World Health Organization (WHO) and global data, cases of human rabies predominantly affect children and the older population (22, 41, 42). World Rabies Day has proven to be highly effective in public education efforts. In response, Shanghai prioritizes scientific popularization during key periods and targets key populations. Municipal and district Animal Disease Prevention and Control Centers organize annual awareness campaigns on rabies and other zoonoses through diverse channels including communities, schools, and online platforms (2, 34). Furthermore, we vigorously promote the concept of responsible pet ownership. This initiative aims to strengthen owners’ responsibilities and ultimately prevent the generation of free-roaming animals. During science outreach events, cultural and creative products, popular science books, brochures, and other materials are distributed to citizens. Meanwhile, the Centers regularly released epidemic prevention tips and educational videos via WeChat’s official account.

For personnel such as veterinarians, centers conduct regular technical training sessions (2, 34). According to the WHO recommendations, pre-exposure prophylaxis (PrEP) is recommended for individuals at a high-risk of rabies exposure (28, 43). Accordingly, veterinarians are required to receive annual PrEP at vaccination points in Shanghai (2, 34).

## 3 Challenges and perspective

Since the enactment of the Dog Management Regulations in 2011, Shanghai has made significant progress in rabies control. The incidence of both canine and human rabies has remained consistently low. Notably, despite over 100,000 annual medical visits for dog bites, human rabies cases have remained in single digits since 2011 (2, 33). However, control targets have not been fully met, and current strategies have overlooked certain transmission risks. To achieve the goal of “Zero human deaths from dog-mediated rabies by 2030,” additional multifaceted interventions are urgently needed.

Although the seropositivity rate exceeds 80% among household dogs (including rural and breeding-farm dogs), rates among free-roaming animals are much lower—14.38% for free-roaming cats and 17.7% for free-roaming dogs (2, 37). As a result, herd immunity in domestic animal populations falls short of WHO recommendations. Recent samples from bite incidents confirm free-roaming dogs as the primary rabies carriers in Shanghai. Mass dog vaccination is widely recognized as the principal method for achieving vaccination coverage of 70% or higher in regions where rabies is transmitted by dogs (2, 13). The lack of an immune barrier in free-roaming animals positions them as a dominant source of transmission. Enhancing the rabies vaccination coverage for free-roaming dogs and cats will be a top priority in Shanghai’s future rabies control policies. Although oral rabies vaccines (ORV) have been effective in wildlife, none are approved for use in dogs or cats (44). Despite its importance for expanding immunization coverage among free-roaming animals, accelerating ORV development and approval remains complex and protracted.

Based on the global framework outlined in “Dog vaccination – barriers and solutions,” Shanghai has demonstrated notable successes in several areas aligned with mass dog vaccination best practices. The city conducts extensive public education campaigns through community events and strictly enforces cold chain management standards for vaccines at vaccination points, effectively addressing barriers related to public awareness and vaccine quality. However, Shanghai still faces significant challenges corresponding to key barriers identified in the guide. A major gap is the lack of accurate dog population data, which falls under Barrier 1: Lacking data on dog population size. Without reliable estimates, vaccination planning, resource allocation, and coverage assessment remain imprecise, hindering effective campaign strategy and evaluation. Additionally, Shanghai struggles with Barrier 7: Insufficient trained personnel to manage vaccination campaigns and conduct vaccinations. This is particularly evident in the limited capacity to vaccinate free-roaming dogs, leading to uneven coverage and undermining herd immunity. The shortage of staff from both the Animal Disease Control Center directly affects the implementation of free-roaming animal vaccination programs, leaving a critical gap in rabies control (45). Moreover, unlicensed pet hospitals, supply stores, and grooming salons—not designated vaccination sites—have illegally administered rabies vaccines to unregistered pets. Some residents also self-administered vaccines bought online (33). These practices increased during the COVID-19 pandemic due to limited access to licensed clinics (3, 32, 46, 47). Improper storage and incorrect administration compromise immunization effectiveness. Stronger oversight of vaccine distribution is needed, including prohibiting sales to unauthorized facilities.

Consequently, enhancing vaccination rates continues to depend on a multi-faceted approach. Veterinary hospitals can be mobilized to form mobile vaccination teams that proactively conduct immunization in areas where free-roaming animals are commonly found. Meanwhile, the Animal Disease Control Center could provide free rabies vaccines to support their efforts. For long-term management, it is essential to begin with a baseline survey to assess the population size and vaccination status of free-roaming animals. Digital technologies should be fully utilized to establish electronic immunization records, creating a unique QR code or ID for each animal. Regular sampling to monitor antibody levels should be carried out, followed by timely revaccination when necessary. Additionally, strengthened multi-department collaboration is crucial. The Agricultural and Rural Affairs Commission should take the lead in providing immunization services and the safe disposal of infected or deceased animals. Public security authorities must enforce stricter pet ownership regulations, impose severe penalties for pet abandonment, address the root causes of free-roaming animal proliferation, and likewise impose severe penalties on hospitals or institutions that violate vaccination regulations. Urban management departments can support through routine patrols and timely reporting of free-roaming animal sightings, while local communities assume responsibility for managing free-roaming animals within their respective areas.

Once herd immunization coverage reaches 70%, containing the dog population can reduce its mobility, thereby helping to maintain high vaccination coverage. In Shanghai’s TNR programs for cats, females are prioritized for spaying. Additionally, ear-tipping is performed on the left ear for males and the right for females to easily identify neutered individuals. Public surveys show broad support for community cat coexistence and the use of TNR programs (38–40). To sustain TNR efforts, collaboration—including government funding, media advocacy, and community engagement—is essential (38, 40). Simultaneously, enforcement agencies should strengthen capture and shelter management of free-roaming dogs. Currently, Shanghai’s management of free-roaming dogs primarily relies on shelter intake, and TNR have not yet been implemented. Building on successful cat strategies, TNR and adoption programs could be expanded to dogs. While not a core component of rabies control, TNR effectively curbs the free-roaming dog population, serving as an indirect measure to manage the disease. Governments should maintain a clear distinction in funding between rabies vaccination and TNR initiatives, ensuring that the central objective of mass dog vaccination remains the strategic focus (48).

In the area of rabies surveillance for dogs and cats, our center annually assigns sampling tasks to designated immunization points, conducting active monitoring for rabies and other zoonotic diseases among domestic pet dogs and cats presented at veterinary clinics. For sheltered animals, we collaborate with Public Security Bureau shelters and district dog management facilities to regularly collect serum samples for active surveillance. However, the surveillance system remains weak for free-roaming dogs and cats, where monitoring typically shifts to a passive approach only after human bite incidents occur. Following such cases, our center conducts risk assessments of the dogs involved, samples any suspected rabid animals, and submits specimens to the National Rabies Reference Laboratory for confirmation. Meanwhile, other dogs from the same social group are captured and placed under observation. Dogs that test positive are humanely culled, while those testing negative are either returned to their owners or released at the original location. The current surveillance gap for free-roaming animals represents a critical weakness in Shanghai’s—and indeed China’s—rabies control framework. Future policy development should prioritize collaboration with neighborhood committees to expand disease surveillance to include the free-roaming dog and cat population. A collaborative mechanism can be established through WeChat mini-programs to engage volunteers and residents in monitoring free-roaming animals for rabies. This platform would facilitate a streamlined process where public reports of suspicious animal locations are directly forwarded to specialized staff from the Animal Disease Control Center and Public Security Bureau for capture and testing.

In countries where canine rabies has been eliminated, reintroduction might occur via wildlife (5–7, 24–29, 49). In China, Jilin reported a canine infection from a bat-origin rabies virus, while Inner Mongolia and Xinjiang documented fox-derived virus transmission to dogs and other animals (8, 50). The potential for rabies reintroduction from wild animals cannot be overlooked, and as such, a vaccination coverage of over 70% must be maintained in domestic dogs and cats even after rabies elimination. Critically, if vaccination coverage in the free-roaming population falls below 20%, efforts to control the virus in wildlife and the development of ORV become largely irrelevant, as the risk of spillover from dogs will be unacceptably high (10, 24, 27). Expanded wildlife monitoring—such as of raccoon dogs—is meaningful in Shanghai to prevent cross-species transmission. Presently, Shanghai is in the early stages of acknowledging the rabies threat from wildlife, with current countermeasures primarily consisting of annual raccoon dog population surveys conducted by volunteers. This initial step paves the way for a more robust approach: scaling up by incorporating disease monitoring into the community-based wildlife census and initiating preventive ORV baiting in identified wildlife hotspots.

Rabies also poses cross-border transmission risks. Europe has recorded multiple imported cases, including a resurgence in Poland 17 years after achieving rabies-free status (51–53). Similar cases have occurred in China (9). With growing pet mobility via cabin access policies, preventing transboundary spread is increasingly urgent (12, 54). Customs authorities should strengthen quarantine procedures and require valid rabies vaccination certificates for imported pets. Establishing a cross-border wildlife surveillance network would strengthen early warning in border regions.

Over the past two decades, locally acquired human rabies cases in Shanghai have been concentrated in suburban districts. Over 80% of cases occurred in males, and more than 40% involved individuals aged 50 or older. Critically, over 85% of infected patients did not receive PEP (21). Over 95% of human rabies cases in China occur in rural areas. The declining proportion of pediatric patients reflects the success of school-based education programs. Despite this, PEP utilization remains low, emphasizing the need for expanded public education, especially in rural communities (18).

Additionally, rabies vaccines and immunoglobulins are generally excluded from China’s medical insurance, creating financial barriers to timely PEP access (55). Future efforts should integrate rabies biologics into insurance coverage and standardize dog-bite injury management to improve access and treatment outcomes.

Addressing implementation challenges will also require timely updates to the Dog Management Regulations. Under the One Health framework, rabies prevention demands multisectoral collaboration beyond agricultural departments. Sustainable policies must integrate medical, veterinary, environmental, and law enforcement sectors. This coordinated approach is essential to reaching the national goal of eliminating dog-mediated human rabies in China by 2030.

## 4 Conclusion

Shanghai’s comprehensive and multi-faceted approach to rabies control has yielded significant successes over the past decade, demonstrating that strong political commitment, systematic policy implementation, and continuous monitoring can drastically reduce the incidence of both canine and human rabies incidence. The integration of compulsory dog registration and vaccination, expanded access to immunization services—especially in suburban and rural areas—standardized management of vaccination points, and targeted public awareness campaigns have collectively contributed to sustaining low human rabies case numbers, despite high annual bite injury incidence.

However, critical gaps remain. The low immunization coverage among free-roaming animals (seropositivity <18%) poses a major risk, as free-roaming animals are identified as the primary source of rabies transmission in Shanghai. Other challenges include illegal vaccine administration by unlicensed facilities, potential wildlife-mediated transmission, cross-border reintroduction risks, low PEP uptake in rural and older population, and financial barriers to PEP access. To achieve the national goal of “Zero by 2030,” Shanghai must adopt a more integrated and expansive One Health approach.

Building on our existing capacities in disease surveillance and public health education, our center should prioritize conducting a comprehensive survey of Shanghai’s dog population to obtain accurate data on rabies vaccination coverage for both dogs and cats. We will strengthen collaborations with communities, veterinary hospitals, and veterinary schools to extend rabies vaccination to as many household and free-roaming animals as possible. Leveraging previous TNR pilot experiences, we will continue promoting TNR programs for both cats and dogs. In the long term, strategic partnerships with the Shanghai Veterinary Research Institute and the Shanghai Academy of Agricultural Sciences could be established to develop ORV for dogs and cats—a game-changing solution that could significantly enhance immunization coverage among free-roaming animal populations.

Shanghai’s experience offers a valuable model for other highly populated urban centers in China and internationally, particularly in regions with high rabies incidence. Its successes highlight the importance of dedicated policy frameworks, accessible services, and public engagement, while the existing challenges emphasize the need for inclusive, well-funded, and collaborative strategies that address all transmission pathways and populations at risk.
